# Tick Control in a Connected World: Challenges, Solutions, and Public Policy from a United States Border Perspective

**DOI:** 10.3390/tropicalmed7110388

**Published:** 2022-11-20

**Authors:** Hannah S. Tiffin, Edwin G. Rajotte, Joyce M. Sakamoto, Erika T. Machtinger

**Affiliations:** Department of Entomology, Pennsylvania State University, University Park, PA 16802, USA

**Keywords:** climate change, global health, globalization, *Haemaphysalis longicornis*, *Ixodes scapularis*, Lyme disease, One Health, *Rhipicephalus*, tick control, zoonosis

## Abstract

Ticks are able to transmit the highest number of pathogen species of any blood-feeding arthropod and represent a growing threat to public health and agricultural systems worldwide. While there are numerous and varied causes and effects of changes to tick-borne disease (re)emergence, three primary challenges to tick control were identified in this review from a U.S. borders perspective. (1) Climate change is implicated in current and future alterations to geographic ranges and population densities of tick species, pathogens they can transmit, and their host and reservoir species, as highlighted by *Ixodes scapularis* and its expansion across southern Canada. (2) Modern technological advances have created an increasingly interconnected world, contributing to an increase in invasive tick species introductions through the increased speed and frequency of trade and travel. The introduction of the invasive *Haemaphysalis longicornis* in the eastern U.S. exemplifies the challenges with control in a highly interconnected world. (3) Lastly, while not a new challenge, differences in disease surveillance, control, and management strategies in bordering countries remains a critical challenge in managing ticks and tick-borne diseases. International inter-agency collaborations along the U.S.–Mexico border have been critical in control and mitigation of cattle fever ticks (*Rhipicephalus* spp.) and highlight the need for continued collaboration and research into integrated tick management strategies. These case studies were used to identify challenges and opportunities for tick control and mitigation efforts through a One Health framework.

## 1. Introduction

In recent decades, reported cases and geographic shifts of (re)emerging vector-borne diseases have been progressively increasing, due in part to rapid urbanization, changes in land use, globalization leading to increased trade and travel, and the changing global climate [1]. These factors are also increasing human exposure to animal reservoirs and arthropod vectors, including increased transmission of pathogens to naïve human populations [1,2]. Ticks transmit the highest number of pathogen species to vertebrates of any blood-feeding arthropod and are a growing threat to public health and agricultural systems worldwide [3,4].

In the United States nearly 95% of reported vector-borne diseases are transmitted by ticks [5]. Of the almost 30 different identified tick-borne diseases in the Western Hemisphere, 12 are considered current and emerging threats to human health in the U.S. [5,6]. Lyme disease is the most frequently reported vector-borne disease in the U.S., with approximately 30,000 confirmed annual cases but upwards of 400,000 individuals that receive treatment for Lyme disease annually [7,8]. The continued expansion of the primary vector, *Ixodes scapularis* (blacklegged tick), into new habitats and geographic regions is concerning [9]. The future poses challenges to tick-borne disease management and predicting the challenges and mitigation strategies is difficult.

One such challenge is climate change, which is implicated in current and future alterations to geographic ranges and population densities of several tick species of medical and veterinary importance [10]. High infestation burdens and associated mortalities due to *Dermacentor albipictus* (winter tick) on moose (*Alces alces*) and other cervids and the range expansion of *I. scapularis* are both partially attributed to the effects of climate change [11,12,13,14,15]. Warmer temperatures, particularly warmer winters, are hypothesized to be a leading factor associated with *I. scapularis* range expansions across eastern and central Canadian provinces [16]. Global climate change will affect tick biology and the hosts and reservoirs involved in tick-borne disease cycles [17,18]. Not only are the vectors and pathogens affected by climate change, but they are affected differentially leading to nonlinear responses and new relationships among vectors, pathogens, and hosts [19].

In addition to transmitting disease-causing pathogens, ticks can also cause morbidity and mortality through painful bites, inflammation, increased stress, inducing tick paralysis and bite-associated meat allergies, toxicosis, or even exsanguination in animals [20]. High tick infestations have led to epizootics in certain ecosystems, with climate change-driven warmer, milder winters often implicated as a principal factor in increased tick overwintering survival [18]. “Ghost moose” have become a banner species for this increase in high parasite loads, with *D. albipictus* infestations on moose so severe that they can result in severe anemia, decreased fecundity in cows, and mortality [21,22].

Another challenge is that modern technological advances have created an increasingly interconnected world. The speed and frequency of trade and travel have contributed to an increase in exotic and invasive tick species introductions [23,24]. The recently detected invasive species to the continental U.S., *Haemaphysalis longicornis* (longhorned tick), is another species with a propensity for high tick burdens on parasitized animals, particularly cattle, and is of medical and veterinary importance due to this aggregation behavior, capacity for parthenogenetic reproduction, and pathogen transmission potential [25,26,27,28]. The longhorned tick has been introduced and proliferated in numerous new regions worldwide and requires intensive surveillance as well as rapid identification and control measures [25,29,30,31,32].

While not a new challenge, effective and consistent disease surveillance, control, and management strategies along shared geographic borders remain a critical challenge in managing ticks and tick-borne diseases, particularly with increased movement across borders due to globalization [23,33,34]. This movement is also fueled by increased meat and dairy consumption and higher demand for livestock trade, increasing the importance of management of transboundary animal diseases and their respective vector species [33,34]. For example, cattle fever ticks (*Rhipicephalus* spp.) are important endemic and invasive ectoparasites of cattle in numerous regions worldwide and can result in substantial welfare concerns and economic losses to the cattle industry [35]. Cattle fever ticks, vectors of the causative agents of bovine babesiosis, are endemic throughout many regions of Mexico [36,37]. This not only affects Mexico’s cattle herds but threatens the re-introduction and re-establishment of cattle fever ticks into the southern U.S. through cattle trade and the movement of wildlife alternate hosts across the Mexico–U.S. border [38,39,40]. This system exemplifies the challenges of implementing effective management and control strategies in disease systems that share common geography across international borders [33,41].

**Figure 1:**
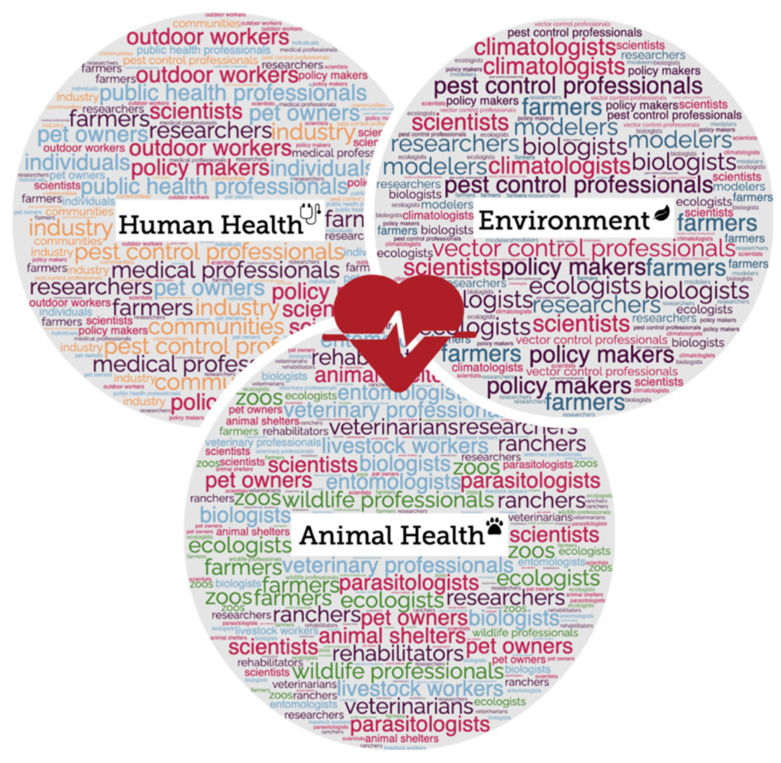
A One Health approach ensures collaboration among the three health sectors—human, animal, and environmental health. Tick control requires collaborative strategies to mitigate disease risk to humans and animals and will require dynamic planning at the agency, community, and individual levels, as highlighted by the examples in this diagram (figure created in the Mind the Graph platform, www.mindthegraph.com, Cactus Communications and Wordclouds.com).

The complex epidemiology and ecology associated with tick-borne diseases presents another challenge. Many tick species prefer one host species but will opportunistically feed on a variety of species, and many hard ticks require two to three different hosts to complete their life cycle [6]. This not only complicates which host species are affected, but also which pathogens may be acquired from these different hosts and transferred to other species by a given tick [10]. A One Health approach, which acknowledges the interconnected nature of human, animal, and environmental health and promotes collaborations between these sectors, is required to successfully manage vector-borne and zoonotic diseases [42,43,44]. This interdisciplinary approach will be paramount for successful tick control and tick-borne disease mitigation strategies and it is imperative for medical and veterinary professionals, policy-makers, and researchers to collaborate to better understand how tick-borne pathogens are moved in the environment and how they may manifest in disease in different host species [45] (Figure 1). While these efforts can be led by the scientific community, input and collaboration from scientists and non-scientists alike will be necessary to identify and implement effective long-term solutions.

**Figure 2:**
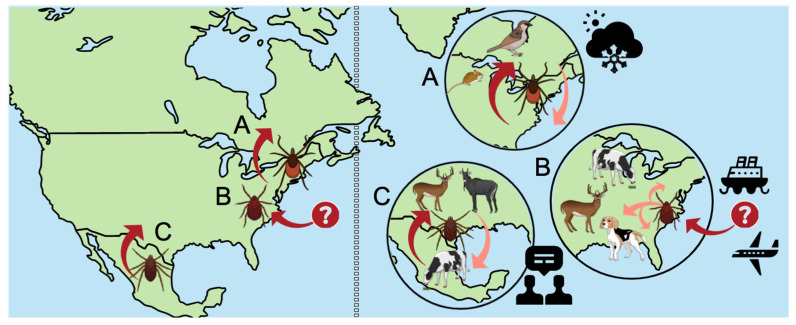
While many challenges remain to tick control, the three identified in this review as the primary challenges in a globalized world are: (**A**) Global climate change, with a case study of *Ixodes scapularis* range expansion from the northeastern U.S. into southern Canada, correlated with warmer and milder winters and tick dispersal associated with migratory birds and range expansion of the white-footed mouse; (**B**) the invasive tick *Haemaphysalis longicornis* introduction and range expansion in the continental U.S., while the initial introduction is unknown it is likely related to trade and travel with a broad host range facilitating regional dispersal; and (**C**) political sovereignty and shared geography between bordering countries, highlighted by the collaborations between the U.S. and Mexico at the southern border to reduce movement of cattle fever ticks (*Rhipicephalus* spp.) through cattle trade between the bordering countries but complicated by tick dispersal via suitable wildlife hosts (primarily white-tailed deer and nilgai antelop—an introduced species) (figure created in the Mind the Graph platform, www.mindthegraph.com, Cactus Communications.

The objectives of this review are to (1) identify the major modern challenges to tick control and (2) develop best management strategies for tick control given these challenges. The following three conditions were identified as the primary challenges to tick control given our interconnected world: (1) global climate change, (2) globalization of trade and travel, and (3) permeable political borders. To further explore these conditions, three case studies from a U.S. borders perspective were used to highlight the challenges and recommendations for tick control in an interconnected world (Figure 2).

## 2. Case Studies Highlighting Challenges to Tick Control

### 2.1. Global Climate Change: Ixodes scapularis Range Expansion into Canada

Lyme disease is the most commonly reported vector-borne disease in the Northern Hemisphere [46]. The causative agent, *Borrelia burgdorferi* sensu stricto (hereafter referred to as *B. burgdorferi*), is most commonly transmitted by *I. scapularis*, which is endemic in the northeastern United States. However, the recent expansion of *I. scapularis* into central and eastern Canada has raised concerns about further spread of the tick vector and cases of Lyme disease, particularly given the effects of global climate change. It is predicted that climate change will create more suitable habitats for the blacklegged tick, in areas previously unsuitable for this species [46,47].

Passive surveillance and identification of *I. scapularis* has occurred in Canada since the early 1990s [48]. However, prior to 1997, only one established population of *I. scapularis* had been documented in Canada [46]. Since then, populations of *I. scapularis* have been expanding their ranges across southern and central Canada and have been found in higher densities in certain locales [49]. Areas with increased densities are attributed to milder temperatures and decreased precipitation related to climate change [50,51]. Given the current climate projections, it is likely that the range of *I. scapularis* will continue to expand and new locations will soon be affected by the tick and its transmissible pathogens [13].

Climate change will not only affect *I. scapularis* survival and range expansion, but the ranges of host and reservoir species as well [17]. Moreover, the relationship between vector and pathogen may also change [19]. This further complicates predictions of *I. scapularis* range expansion and predicted Lyme disease risk maps [46]. Given the importance of small mammals for the Lyme disease cycle [52,53], it is imperative to consider the current and predicted ranges of suitable hosts and reservoirs for *B. burgdorferi* [17,18,54,55]. An estimated risk index of *B. burgdorferi* occurrence in Canada indicated that pathogen hotspots were highly dependent on the presence and abundance of the white-footed mouse (*Peromyscus leucopus*), particularly in association with warmer winter temperatures [17]. Historical records and models indicate northward range expansions of the white-footed mouse, with ranges predicted to continue expanding across southern Canada [56].

Distribution modeling combined with classical surveillance can predict and verify new areas of suitability for targeted control and continued surveillance needs [57]. By integrating targeted surveillance results into models, more accurate predictions are attainable [58]. This feedback loop can provide high-quality surveillance insights for detecting new populations of *I. scapularis* as well as directing control strategies to areas at greatest risk for *I. scapularis* population establishment [59]. When long-term surveillance records and modeling tools were used in tandem, temperature was found to be the principal driver of *I. scapularis* range expansion, with fewer freezing days associated with tick range expansion and establishment [59]. Using models, classical tick monitoring methods (e.g., dragging with a white cloth over standardized distances to estimate abundance of questing ticks in the environment), and on-host surveillance strategies in tandem can direct surveillance efforts and funnel resources to areas predicted to be most suitable for tick range expansion [47,57,59,60].

The Canadian government instituted several critical components into their tick-borne disease mitigation programs in the early 1990s, which aided in detecting the expansion of *I. scapularis* in real-time and consequently expanding their surveillance programs [47]. While reported cases of Lyme disease are still much lower than those in the Northeast U.S., public education campaigns to raise awareness of the risks and protective measures available have created a strong passive surveillance program for the public, veterinarians, and medical professionals to submit ticks to participating laboratories for identification and testing for nearly three decades [46,48,51]. Public education campaigns are critical to building a successful disease mitigation program [23,47,61]. The expansion of *I. scapularis* into new geographic regions exemplifies the numerous challenges associated with a warming climate, as well as the potential for proactive mitigation strategies.

### 2.2. Globalization: Haemaphysalis longicornis Introduction into the Continental U.S.

*Haemaphysalis longicornis*, the longhorned tick, has a long history of misidentification, introduction, and eventual establishment outside of its native range [29]. Native to eastern Asia, this species was likely introduced to Australia, New Zealand, and other Pacific Islands through the movement of infested cattle [29]. It has since spread and become established in several regions throughout Asia and Oceania and most recently, in several states in the continental U.S. [25,27,28,29,62,63]. Its ability to successfully become established in numerous different climates is likely due to its broad host range, including livestock species such as cattle and sheep, domestic animals, and wildlife species, as well as its ability to reproduce parthenogenetically (asexually) [29,30,62].

*Haemaphysalis longicornis* poses both medical and veterinary risks as it can transmit a variety of pathogens in its native and exotic ranges. In its native East Asia range, *H. longicornis* transmits two potentially lethal pathogens to humans: severe fever with thrombocytopenia syndrome virus (SFTSV) [64,65,66], and *Rickettsia japonica* (the etiological agent of Japanese spotted fever) [67,68,69]. Livestock are particularly vulnerable to *H. longicornis* infestations. Even if not infected with one of the many pathogens this tick can transmit, they can become stressed or even die of toxicosis or exsanguination due to the high tick burdens that can propagate on these hosts.

The first population of *H. longicornis* in the continental United States was identified in 2017 on a sheep in New Jersey [25]. With all life-stages of the tick present on the animal and no history of travel outside of the U.S., an established population of *H. longicornis* was suspected [25]. In 2018, enhanced surveillance and detection methods in response to the initial infestation detected *H. longicornis* populations at the initial site of infestation, in additional counties in New Jersey, as well as in seven other eastern states: Connecticut, Maryland, New York, North Carolina, Pennsylvania, Virginia, and West Virginia, and as far west as Arkansas [28]. Additionally, reexamination of archived samples identified *H. longicornis* in samples collected from a white-tailed deer in 2010 in West Virginia and a domestic dog in 2013 in New Jersey, indicating the establishment of this tick prior to the discovery of the infested sheep in New Jersey in 2017 [28]. As of August 2022, *H. longicornis* local populations have been identified in 17 states: Arkansas, Connecticut, Delaware, Georgia, Kentucky, Maryland, Missouri, New Jersey, New York, North Carolina, Ohio, Pennsylvania, Rhode Island, South Carolina, Tennessee, Virginia, and West Virginia [70].

*Haemaphysalis longicornis* has a broad host range and in the U.S. has been found parasitizing domestic animals, livestock and equine species, and numerous mammalian wildlife and avian species [62,71,72]. This broad host range combined with its parthenogenetic ability has made *H. longicornis* a difficult species to control. However, even though *H. longicornis* has been found on humans in the U.S., there have been few reported bites [26]. Additionally, a recent study found that *H. longicornis* preferred hair from domestic cats and dogs and white-tailed deer and avoided hair from white-footed mice and humans [73]. This may indicate a preference for medium- and large-bodied mammalian hosts in the U.S. [72].

Though this species is a new threat in the U.S., there have already been reports of cattle mortality due to *H. longicornis* infestation [74]. Additionally, while the extent of pathogen presence in U.S., *H. longicornis* populations, is unknown, *Theileria orientalis* Ikeda subtype has been implicated as the cause of death of several cattle in a Virginia farm [75,76], and is effectively transmitted to cattle by U.S.-collected *H. longicornis* in lab experiments [77]. This is highly concerning as *T. orientalis*, particularly the Ikeda subtype, can be extremely pathogenic to livestock [30,78]. While other pathogens have been detected in *H. longicornis*, it is currently unknown if this tick can successfully transmit these pathogens, including Bourbon virus, *Anaplasma*, *Borrelia*, and *Ehrlichia* species [27,79]. A recent study found that *H. longicornis* was not able to successfully transmit *B. burgdorferi* B31 strain to mice under laboratory conditions [80], potentially providing some relief to the U.S. Northeast already burdened by high numbers of Lyme disease cases.

*Haemaphysalis longicornis* presents numerous challenges to control, namely its capacity for parthenogenetic reproduction and severe infestations, numerous suitable hosts, and few identifying features to untrained (and trained) personnel [29,30,62]. Of available acaricides, recent research has indicated that commercially available spray and pour-on acaricides were effective against U.S. populations of *H. longicornis* [32]. However, more research is needed to identify other methods to control *H. longicornis* populations and mitigate bite risk, particularly for livestock, as chemical acaricides are currently the primary control method and these pose environmental risks, non-target effects, and the potential for acaricide resistance to develop [81,82].

### 2.3. Policy and Shared Political Borders: Rhipicephalus spp. Tick Management at the Mexico–U.S. Border

The increased global demand for livestock products, particularly dairy and beef, have put additional strain on typical control methods of endo- and ectoparasites in livestock systems [81]. Ticks, in particular, can cause significant animal welfare concerns and economic losses in dairy and cattle operations, with the southern cattle fever tick (*Rhipicephalus microplus*) widely considered to be the most significant economic burden to livestock operations globally [35,83]. Cattle fever ticks can transmit *Babesia* spp., the causative agent of bovine babesiosis, which can result in decreased dairy and meat production, increased abortion rates, and mortality to infected cattle [36].

The two cattle fever tick species of concern in North America are *R.* (*Boophilus*) *microplus* and *R.* (*B.*) *annulatus*, responsible for transmitting *Babesia bovis* and *B. bigemina* [84]. Of note, this particular disease system was the first time transmission between an arthropod vector and mammalian host was discovered [85]. The high costs to cattle producers from cattle fever ticks and bovine babesiosis resulted in a flurry of research on disease mechanisms, control, and plans for eradication [86]. Cattle fever ticks were effectively eradicated from their range in the southern U.S. by 1960; however, *R. microplus* and *R. annulatus* are endemic in many regions throughout Mexico, making eradication and mitigation consistent objectives [33,39,86]. Given the propensity of *R. microplus* for wildlife hosts in addition to cattle, it is likely that cattle fever ticks have been reintroduced into the U.S. on numerous occasions from wildlife hosts moving across the Rio Grande tick eradication quarantine area between southern Texas and northern Mexico [39].

It is estimated that 65% of Mexico’s land area is infested with *R. microplus* and 75% of cattle herds are at risk for bovine babesiosis infection, with the potential total economic losses due to *R. microplus* estimated at over USD 500 million ($US) [36]. While previous control efforts focused on the use of acaricide cattle dips, regulated cattle transport and inspections across the Mexico–US border, and a permanent quarantine zone in southern Texas along the Rio Grande, increased acaricide resistance and wildlife reintroductions of cattle fever ticks across the border have complicated control and eradication efforts [33,39].

To meet these challenges, an international group of livestock producers and stakeholders, researchers, industry representatives, and regulators met in 2019 to discuss current challenges, priorities, and management needs for cattle fever ticks and bovine babesiosis [33]. One of the primary challenges identified during this meeting was the increased reports of acaricide resistance in cattle fever ticks [33]. Concern for the environmental effects of acaricide disposal has also contributed to an increased need for integrated management options for tick control [81,82].

Nearly 50% of countries have documented acaricide resistance in their veterinary relevant tick populations, with many documenting cross-resistance in ticks and other parasitic groups, such as helminths, mites, flies, and lice [87]. While traditional cattle fever tick control has relied heavily on acaricide treatments, there are cattle and landscape management strategies shown to reduce tick infestations as well as optimistic results from rotating acaricides [81,83,88].

While cattle fever ticks have a strong historical association with host-parasite research and innovations, there is still much to learn and develop within this system. Collaborations across entities and international borders will continue to be important in developing integrated management strategies for cattle fever ticks that reduce control and environmental challenges associated with acaricide reliance and resistance [33,61,82]. Management strategies are also in development for wildlife components to these systems, frequently within One Health contexts to account for environmental and human health connections [81,82,89].

## 3. Conclusions

### 3.1. Lessons Learned and Future Needs

**Figure 3:**
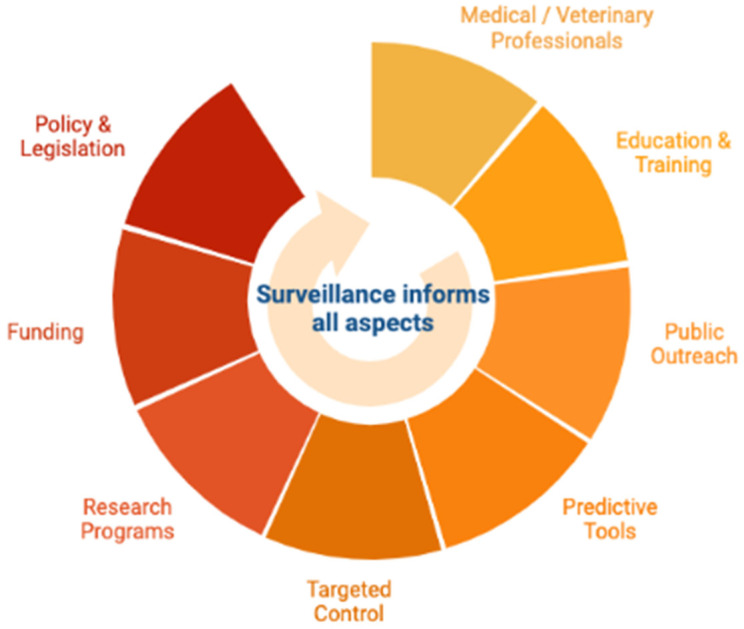
Effective tick control requires continued surveillance and adaptive control, management, and education to fit the current and predicted future needs of the populace (figure created with BioRender.com).

The increasing (re)emergence of numerous zoonotic and vector-borne diseases, particularly related to ticks and tick-borne diseases in the Northern Hemisphere, exemplifies the need for efficient surveillance programs, collaborations between agencies and across borders, the need for continued research on control and mitigation tools, and the importance of public health education campaigns (Figure 3) [33,54,90]. Nothing can highlight the importance of these systems as well as the COVID-19 pandemic, which put the global human health and research networks in the public eye, revealing many weaknesses in the public health infrastructure as well as strengths in scientific collaborations and technological advancements [91,92,93,94].

Models have shown conflicting results on the effects of climate change on tick distributions and tick-borne disease risks, as can be expected with complex systems, and have shown much broader habitat suitability than current species records indicate in many cases [54]. Additionally, macro- and micro-habitats are critically important in interpreting and extrapolating model predictions. For instance, models in Canada have shown the importance of warmer, milder winters in the range expansion of *I. scapularis*. In contrast, models in the northeastern U.S., where Lyme disease is endemic, have not detected a significant effect of weather conditions on tick densities in the following years [95]. There are numerous varied and complex differences between these two systems, but the difference in importance of a critical predictor such as temperature highlights the caution needed for extrapolating results to different habitats, and particularly within policy and control applications. Models used in combination with long-term classical surveillance have been effective in developing control and management strategies in public health contexts, as evidenced by *I. scapularis* range expansions in the U.S. and Canada [54,59,96], as well as within veterinary health contexts, such as in *Rhipicephalus* spp. in cattle-wildlife grazing systems in Kenya [97,98,99,100].

A major caveat to the disease systems discussed herein is the long-term surveillance and research conducted and the resources allocated toward these ends. However, these systems were also chosen for these reasons, to derive insights on surveillance, control, and management strategies that are relevant and applicable for systems with newly discovered diseases or vectors, less research conducted, and/or limited resources available for disease surveillance and mitigation. A primary example of strategies that could lessen resource requirements for surveillance is to focus surveillance efforts on model-predicted locations of highest risk [57,59]. However, many entities may not have the resources available (technology, expertise, personnel, time, or funds) to invest in regional or local level models [47,54]. In addition to the obvious local public health benefits, collaborations to develop models and surveillance methods with local leaders in an increasingly interconnected world benefits the research and public health community-at-large. By employing a One Health framework these collaborations can also extend beyond pressing surveillance and control needs, as evidenced by the working group established between Mexico and U.S. cattle fever tick stakeholders and researchers. This large-scale collaboration has succeeded in establishing best management strategies, research priorities, and control implementation policies for cattle fever tick importation across borders [33]. However, as with most disease systems, this is an ongoing challenge and will continue to require collaboration, additional research to improve control strategies, and continued dialogue on policies and regulations within an integrated One Health context [33,82,89,101]. Additionally, as evidenced by the pressing concerns of acaricide resistance described in the cattle fever ticks case study, there is a persistent need for the development of integrated management tools for tick control outside of chemical applications [83,102]. Other high-priority research endeavors to improve tick control include parasite–host selection and interactions, with on-host selection chronically understudied [103].

While shared borders have historically suffered from shared pests and diseases [24], the globalization of trade and travel has increased the frequency of imported pests and associated diseases to new locations [23,61]. Additionally, increased habitat fragmentation due to urbanization, agricultural intensification, and other land-use changes can increase tick burdens at habitat edges due to complex interactions between wildlife diversity and the availability of anthropogenic food sources for generalist hosts such as white-tailed deer [3]. These spatiotemporal challenges highlight the critical importance of continued and increased surveillance of imported animals at borders, as well as education initiatives for travelers to check for “hitchhikers” while traveling and upon return [23], and for continued research on wildlife and environmental movement of ticks [39,62,72,104,105].

Education is not only important in detecting new ticks at borders, but for public health purposes [47]. Publicly available information is particularly important in regions with emerging ticks and tick-borne diseases as the public is frequently less aware of the risks or the preventative options available to them [106]. However, even while the public is frequently aware of Lyme disease, tick preventative behaviors and control options may not be well understood or employed by the public, indicating a disconnect between disease information and preventative behavior [106]. Even in Lyme disease endemic regions, knowledge of tick identification, tick control and prevention, and tick-borne disease risk is low among the public [106,107]. In cases when survey respondents had a moderate or high knowledge of Lyme disease preventative information, many were still unaware of other tick species and tick-borne diseases present in their geographic region [108]. However, studies quantifying changes in behavior after education campaigns are limited for tick-borne disease systems and is an area warranting additional study to effectively educate the public on tick bite risk and prevention methods [109]. Additionally, to motivate and encourage continued behavioral protective measures, it is critical to convey individual agency in tick bite protection to avoid overwhelm and complacency. Combined with additional research on long-term behavioral changes, research is needed on information delivery methods to empower individuals to reduce tick bite risk. This presents another opportunity for One Health collaborations, to address the biobehavioral, psychological, and health equity aspects to tick-borne disease risk reduction.

### 3.2. Public Policy Recommendations

While ticks and tick-borne diseases have a long history with human and animal health, there is still much to learn. The primary modern challenges identified from a U.S. borders perspective in this review included global climate change, globalization of trade and travel, and the continued control challenges associated with shared political borders. The case studies selected for this review represented systems with long-term research and control activities to best understand the challenges, resources available, and research needs associated with tick control and tick-borne disease risk mitigation.

As evidenced in these systems, active and passive surveillance combined with modeling predictions of habitat suitability of ticks and host species is critical in developing a proactive risk management system. Collaborations using a One Health framework within and across agencies and political borders will only become more important as globalization continues to increase the frequency and speed of trade and travel. Increased emphasis on professional identification training, improved detection of exotic species introductions, as well as improved public education will continue to strengthen tick and tick-borne disease detection, mitigation, and control. Lastly, it is evident in this review that increased research on integrated tick management options as well as wildlife and environmental connections to disease cycles is imperative to better understand and control ticks and tick-borne diseases in an increasingly interconnected world.

## Data Availability

Not applicable.
